# Using XGBoost-SHAP for understanding the ecosystem services trade-off effects and driving mechanisms in ecologically fragile areas

**DOI:** 10.3389/fpls.2025.1552818

**Published:** 2025-04-28

**Authors:** Peiyu Du, Heju Huai, Xiaoyang Wu, Hongjia Wang, Wen Liu, Xiumei Tang

**Affiliations:** ^1^ Information Technology Research Center, Beijing Academy of Agriculture and Forestry Sciences, Beijing, China; ^2^ National Engineering Research Center for Information Technology in Agriculture, Beijing Academy of Agriculture and Forestry Sciences, Beijing, China; ^3^ Coll Resources & Environment, Shandong Agriculture University, Tai An, China

**Keywords:** ecosystem services, trade-offs and synergies, XGBoost-SHAP, driving mechanism, ecologically fragile areas

## Abstract

**Introduction:**

Understanding the spatial and temporal variability of Ecosystem services (ES), along with the trade-offs and synergies among different services, is crucial for effective ecosystem management and sustainable regional development. This study focuses on Wensu, Xinjiang, China, as a case study to address these challenges.

**Methods:**

ES and their trade-offs were systematically assessed from 1990 to 2020. Explainable machine learning models (XGBoost-SHAP) were employed to quantify the nonlinear effects and threshold effects of ES trade-offs, with specific attention to identifying their driving factors.

**Results:**

(1) From 1990 to 2020, water yield (WY) and soil conservation (SC) exhibited an inverted "N"-shaped downward trend in Wensu County: mean annual WY decreased from 22.99 mm to 21.32 mm, and SC per unit area declined from 1440.28 t/km² to 1351.3 t/km². Conversely, windbreak and sand fixation (WS) showed an "N"-shaped increase from 2.32×10⁷ t to 3.11×10⁷ t. Habitat quality (HQ) initially improved then deteriorated, with values of 0.596, 0.603, 0.519, and 0.507 sequentially. (2) Relationships between WY-WS, WY-HQ, WS-HQ, SC-WS, and SC-HQ were primarily tradeoffs, whereas WY-SC interactions were synergistic. Trade-offs for SC-HQ, WY-HQ, and WS-HQ were stronger, while WY-SC trade-offs were weaker. (3) The XGBoost-SHAP model revealed land use type (Land), precipitation (Pre), and temperature (Tem) as dominant drivers of trade-offs, demonstrating nonlinear responses and threshold effects. For instance, WY-SC trade-offs intensified when precipitation exceeded 17 mm, while temperature thresholds governed WY-HQ trade-off/synergy transitions.

**Discussion:**

This study advances the identification of nonlinear and threshold effects in ES trade-off drivers. The model's interpretability in capturing these complexities clarifies the mechanisms underlying ES dynamics. Findings are generalizable to other ecologically vulnerable regions, offering critical insights for ecosystem management and conservation strategies in comparable environments.

## 1 Introduction

ESs are defined as tangible and intangible goods that humans derive from ecosystems (Costanza et al., 1997). These services are critical for human survival, economic sustainability, and natural environmental protection (Bi et al., 2024). In recent years, advances in remote sensing technology and the maturation of relevant theories have shifted research focus from ecosystem structure to ecosystem function, promoting the use of quantitative methods in ES analysis (Bagyaraj et al., 2023). Scholars have conducted extensive research on ES assessment at global and regional levels, developing new indicators and modeling techniques (Gianuca et al., 2024; Marali et al., 2025). However, approximately 60% of the world’s ESs are currently degrading due to the intensifying effects of climate change and increasing human activities (Keesstra et al., 2018). Consequently, research on ES assessment and the determination of the relationship between ESs has become a primary focus of attention for numerous scholars.

Ecosystems are complex and interconnected wholes, and the ESs they provide are of various types with significant differences in spatial distribution. Because human demand for these ESs fluctuates over time and across regions, meaning that changes in one service can affect others. Research on ES relationships in different regions has yielded varying conclusions. Understanding spatial variability and the interrelationships among ESs is crucial for achieving the coordinated development of natural ecosystems and human society. Currently, the relationships among ESs are primarily identified using four main methods: metric description, spatial mapping, scenario analysis, and modeling analysis. Numerous studies (Loiselle et al., 2023; Yuan et al., 2024) have shown that the relationship between ESs is mainly characterized by trade-offs and synergistic relationships of mutual benefit (Yang et al., 2015; Niu et al., 2022). Trade-offs and synergies relationships between different types of ESs are common (Tian et al., 2024), and therefore, the primary goal of ES management is to create a “synergistic optimization” situation by promoting synergies and minimizing trade-offs. In recent years, researchers have identified trade-off synergies between ESs across various scales, from regional to global (Xia et al., 2023). Most scholars have used Spearman’s and Pearson’s correlation coefficients to identify trade-off synergies between pairs of ESs (Qu et al., 2024). However, both correlation methods generally fail to control for the effects of other variables and are sensitive to nonlinear relationships and outliers. This may lead to spurious correlations or inaccurate causal judgments that do not reflect the underlying complexities of the data. In contrast, partial correlation analysis can examine the relationship between two correlated variables while controlling for other factors, allowing for a more objective analysis of the trade-off synergies between two ESs. Therefore, this study uses partial correlation analysis to comprehensively examine the trade-off synergies between ESs. Furthermore, the trade-off relationship is not only reflected in changes between services but also in the non-uniform rate of unidirectional change, which may have a greater impact than the synergistic effect (Lu et al., 2014). The root-mean-square deviation (RMSD) method can consider the interaction between multiple ESs while dealing with the uneven co-directional changes of ESs. It is capable of effectively detecting significant biases in the data and extending the traditional negative correlation trade-off relationship to inhomogeneous rates of spatial variability in magnitude. This compensates for the lack of spatial information and provides a quantifiable indicator for assessing the strength of trade-offs between pairs and multiple ESs (Bradford and D’Amato, 2012).

Quantifying the trade-offs between ESs and clarifying their driving mechanisms is crucial for understanding how trade-offs arise and how to minimize them. (Wang et al., 2021; Shao et al., 2023). Currently, research on ESs is gradually shifting from a focus on inter-service relationships to analyzing the underlying driving mechanisms. In recent years, methods for exploring the drivers of ES trade-off synergistic relationships have primarily included redundancy analysis (Feng et al., 2017), regression analysis (Liu and Tang, 2024), and geographic detector (Wang et al., 2010). Nevertheless, these methods have yet to fully clarify the nonlinear attributes of the drivers, their trajectory of action, and response characteristics (Gao et al., 2021). Therefore, there is an urgent need for a systematic and comprehensive understanding of the driving mechanisms behind the relationships among ESs.

The rapid development of machine learning (ML) has had a profound impact on numerous scientific fields, including ecology. It involves the development of computer algorithms that can be automatically improved through experience, and has demonstrated a wide range of applications and great potential (Recknagel and Staiano, 2019; Doan et al., 2024). ML algorithms extend traditional analytical methods in the field of ESs (Hampton et al., 2013; Dutta and Arhonditsis, 2023) and can efficiently process big data and reveal complex nonlinear relationships, demonstrating strong stability and robustness (Li X, et al., 2024). However, the “black box” nature of ML algorithms poses challenges for explainability. Although ML algorithms improve prediction accuracy, they often overlook a thorough evaluation of spatio-temporal environmental variables and struggle to offer transparent explanations for forecasting changes in ESs (Guidotti et al., 2019).

To address this, explainable ML methods have emerged to enhance model transparency and interpretability. One such method is XGBoost-SHAP, which combines the power of XGBoost, a highly effective gradient boosting model, with SHAP (Shapley Additive Explanations). XGBoost-SHAP is particularly suited for studying ES trade-offs, as it can identify the direction of driving factors and capture their nonlinear responses. This is a significant improvement over traditional methods, which often fail to fully account for the complex interactions that characterize ESs.

Although XGBoost-SHAP has seen success in other fields (Molnar et al., 2020), it has been underutilized in research on ES trade-offs. This study aims to fill this gap by applying XGBoost-SHAP to better understand the nonlinear relationships and driving mechanisms behind ES trade-offs. This approach not only improves prediction accuracy but also offers valuable insights into the complex dynamics that traditional models cannot capture.

Wensu County, located in Aksu Prefecture in the Xinjiang Uygur Autonomous Region of China, serves as a typical study area of the ecological fragility in the northwest region. Much of the existing research focuses on empirical assessments of the vulnerability of ecologically fragile areas, ecological security patterns, ecological industries, and products (Nilsson and Grelsson, 1995; Li L, et al., 2021; Fang et al., 2022). Meanwhile, ESs related to water and soil, along with their complex interrelationships, with the context of the region’s vulnerable ecological baseline, urgently require further investigation. Therefore, research on the trade-off effects and driving mechanisms of ESs in ecologically fragile areas is essential for their management and sustainable development.

ES trade-offs and synergies are key concepts in understanding the complex interactions between human activities and natural ecosystems. In recent years, there has been growing interest in how these interactions vary across different regions, particularly in ecologically fragile areas. These regions face unique challenges such as extreme climate conditions, land degradation, and unsustainable land-use practices. In ecologically fragile areas, critical factors influence the trade-offs and synergies between ESs: Climate stressors, such as precipitation patterns, temperature fluctuations, and seasonal variations, play a dominant role in shaping the interactions between ESs. For example, in arid regions, a slight shift in precipitation levels can trigger significant changes in the availability of water resources and the capacity for soil texture, leading to either synergies or trade-offs depending on thresholds; changes in land use, particularly through urbanization, agriculture, and deforestation, drive trade-offs between ESs by fragmenting habitats and reducing biodiversity. Rapid land conversion can exacerbate service trade-offs, leading to the degradation of services like HQ; in ecologically fragile areas, particularly those with sandy or degraded soils, low water retention exacerbates conflicts between agricultural and ecological needs. The interaction between climate and soil properties can be modeled to assess how thresholds in soil moisture and precipitation affect service trade-offs, providing insights into potential management strategies.

Building on the previous analysis, this study adopts the “element-service-relationship-mechanism” cascade framework to explore the ESs dynamic spatio-temporal correlations and the mechanisms driving trade-offs between key ESs. By analyzing the typical county ecological background and socio-economic conditions in ecologically fragile areas, ESs are estimated, trade-offs and influencing factors are explored, and a practical basis is provided for the protection and restoration of ecologically fragile areas. This study offers new insights into the sustainable development of ES management in ecologically fragile areas. Accordingly, this study selected Wensu County in Xinjiang, China as the research area, with the objective of studying four types of ESs from 1990 to 2020. The research objectives are as follows: (1) Quantify the ESs in Wensu County; (2) Analyze the temporal and spatial changes in ESs in Wensu County and the trade-off and synergy relationships; and (3) Explore the nonlinear and threshold effects of ES trade-offs. The goal is to provide theoretical guidance for the stewardship of ESs, the harmonization of human-land interactions, and the realization of sustainable regional development.

## 2 Materials and methods

### 2.1 Study area

**Figure 1:**
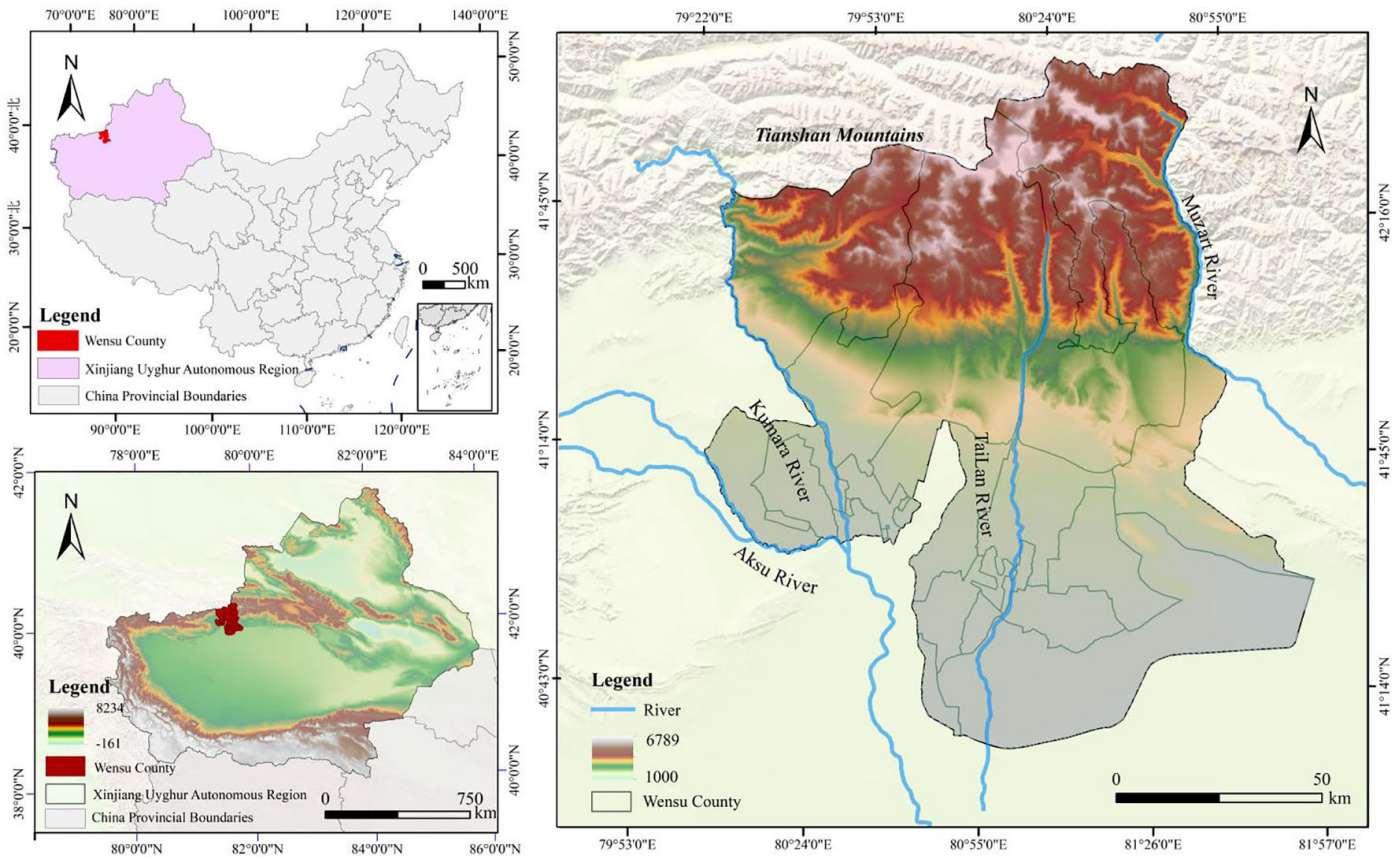
Location map of the study area. Based on the standard map No. GS (2024) 0650 on the website of the Ministry of Natural Resources of the People’s Republic of China, with no boundary modification on the base map.

This study selected Wensu County, Aksu Prefecture, Xinjiang Uygur Autonomous Region, China (**Figure 1**) (40°52’–42°15’N, 79°28’–81°30’E). This region is located in the arid area of the hinterland of the continent, with a total area of 14,569.3 km^2^. It has a continental warm arid climate with an average annual temperature of 10.10°C and an average annual precipitation of 65.4 mm(https://www.geodata.cn). The terrain is varied, with the land rising to the north and falling to the south, sloping from northwest to southeast. The wind is strong and the sand is heavy, the soil is poor, and the problems of drought and land desertification are prominent. As a typical ecologically fragile area in the northwest, it provides an ideal setting for studying the synergistic relationships and trade-offs in ESs. The complex interactions between ecosystem components, coupled with the region’s vulnerability to environmental stressors, make it a key area for research aimed at understanding the interrelationships of ESs and identifying effective strategies for sustainable management and protection.

### 2.2 Materials

This study used multiple data sources, including land use data, remotely sensed imagery, meteorological data, digital elevation models, soil datasets, and normalized vegetation indices. In ArcGIS, the data from different sources were resampled and projected, and finally unified and projected to WGS_1984_Albers. The relevant data sources are listed in **Table 1**.

**Table 1:** Data source.

Data name	Data resource	Type	Accuracy
Administrative boundary	Data Centre for Resource and Environmental Sciences, Chinese Academy of Sciences (http://www.resdc.cn)	Vector	1:100,000
LULC		Raster	30m
GDP		Raster	1km
Landforms		Raster	1km
DEM	Geospatial data cloud (https://www.gscloud.cn)	Raster	90m
Vegetation Coverage	National Tibetan Plateau Science Data Center (http://data.tpdc.ac.cn)	Raster	1km
Snow cover data		Raster	500m
Meteorological data	National Earth System Science Data Center (https://www.geodata.cn)	Raster	1km
	China Meteorological Data Sharing Network (https://data.cma.cn)	Raster	1km
Soil data	Harmonized World Soil Database (HWSD) (https://www.fao.org)	Raster	1km
NDVI	MODIS MOD13Q1 product (https://ladsweb.nascom.nasa.gov)	Raster	1km
Population Density	https://www.worldpop.org/	Raster	1km
Road and River data	Open Street Map (https://www.openstreetmap.org/)	Vector	1:100,000

### 2.3 Methods

#### 2.3.1 Research framework

**Figure 2:**
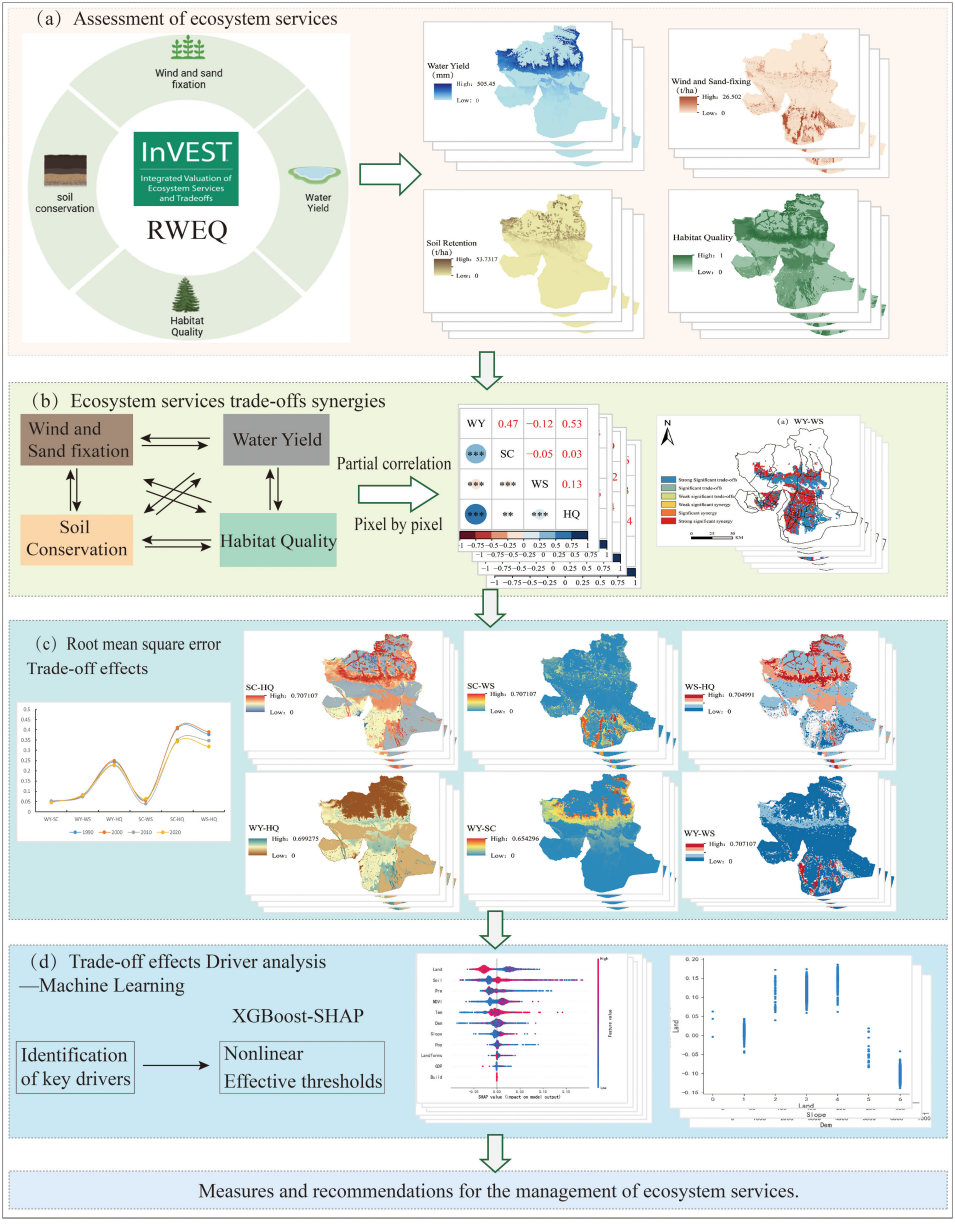
Technical process. **(a)** Assessment of ESs, **(b)** ESs trade-offs synergies, **(c)** Root mean square error trade-off effects, **(d)** Trade-off effects driver analysis - machine learning.

This study focuses on Wensu County, assessing regional ESs and their trade-off synergies, and analyzing the nonlinear characteristics and threshold effects of these trade-offs using explainable ML models. **Figure 2** illustrates the comprehensive technical process. Firstly, based on existing research findings, as well as the natural environment and social conditions of the study area, the following indicators were selected to assess the ecosystem functions of Wensu County from 1990 to 2020: water yield (WY), soil conservation (SC), windbreak and sand fixation (WS) and habitat quality (HQ) were selected for their relevance to the vulnerable ecosystems in Wensu County. Among these, the WY is one of the most important ESs in arid and semi-arid regions. WY is essential for agriculture, industry, hydropower generation, and daily activities, playing a pivotal role in the sustainable development of ESs; SC, as an important ES, is crucial for preventing land degradation in the region and plays a significant role in ecological development and the formulation of protection measures; Wensu County is situated in the Xinjiang Uygur Autonomous Region, which has the highest concentration and largest area of desertified land in China. The question of how to control and combat desertification in Xinjiang has consistently been a topic of significant research interest; HQ is an important indicator for assessing the ability and potential of the environment to provide suitable conditions for the survival and reproduction of organisms. Secondly, a partial correlation analysis was employed to calculate the time series and determine the correlation coefficient between pairs of ESs on an annual basis. This method controls for the influence of other variables, allowing the direct relationship between the two variables to be revealed and the significance of the relationship between ESs to be tested using a t-test (Liu et al., 2022). A positive correlation coefficient between ESs, when passing the significance test, indicates a synergistic relationship, while a negative value indicates a trade-off. Subsequently, the RMSD (Jia et al., 2022; Qiao et al., 2024) is employed to quantify the strength of trade-offs between multiple ESs. A higher value indicates a greater trade-off, allowing the degree of trade-offs between different ESs to be assessed. This method extends the traditional negative correlation trade-off relationship, compensates for the lack of spatial information, and provides an effective, quantifiable indicator for evaluating the strength of trade-offs in ESs. Finally, 10 representative factors were selected for the explainable ML method to analyze the driving factors influencing the trade-offs between ESs. These factors included elevation (Dem), slope (Slope), Normalized Difference Vegetation Index (NDVI), precipitation (Pre), temperature (Tem), soil type (Soil), landform type (Landforms), land use type (Land), population density (Pop), and economic factor (GDP). In contrast to earlier methodologies that have encountered the problem of inadequate interpretation when quantifying the drivers of ES trade-offs through linear fitting or geodetector models, the SHAP method is capable of providing unambiguous explanations for a range of “black box” models. The combination of the XGBoost model with the SHAP method allows for the determination of the direction of movement of the driving factors, the identification of the nonlinear relationship between the ES trade-off effect and the driving factors, and the analysis of the independent impact of each driving factor on the ES trade-off effect. This constitutes the inaugural application of the model in ecologically fragile areas. Based on the research results, measures and related recommendations for the management of regional ESs are proposed.

#### 2.3.2 Quantification of ESs

The methodology for quantifying ESs is shown in **Table 2** (see “Equation 1–16”). 

**Table 2:** ESs model.

Index	Formula		Formula specification
Habitat Quality (Luo et al., 2023, Li Q, et al., 2024)	$D_{xj}=\sum_{r=1}^{R}\sum_{y=1}^{Y_{r}}(\frac{W_{r}}{\sum_{r=1}^{R}W_{r}})r_{y}i_{rxy}\beta_{x}S_{jr}$	(1)	Where *D_xj_* is the habitat degradation degree of grid *x* in land use type *j*; *r* refers to the stressor, *R* is the number of stressors; *y* refers to the number of grids for stressor *r*, *Y_r_* is the number of grids occupied by stressor *r*; *W_r_* is the weight of the stressor; *r_y_* is the threat intensity; β *_x_* is the threat accessibility level of the habitat grid; *S_jr_* is the sensitivity of habitat type *j* to the stressor; *i_rxy_* is the impact of the stressor in grid *y* on grid *x*.
	$Q_{xj}=H_{j}(1-\frac{D_{xj}^{x}}{D_{xj}^{z}+k^{z}})$	(2)	Where *Q_xy_* is the HQ level of grid *x* in land use type *j*; *H_j_* is the habitat suitability of habitat type *j*; and *z* and *k* use the default parameters of the model.
Soil Conservation (Shen et al., 2020)	SC=R×K×L×S×(1−C×P)	(3)	Where *SC* is SC (t·hm^-2^· yr^-1^), *R* is the erosivity of precipitation [MJ·mm/(hm^-2^·h·a)], *K* is the erodibility of soil [t·hm^2^/(hm^2^·MJ·mm)], *LS* is the terrain factor, *C* is the vegetation management factor, and *P* is the soil and water conservation measure factor.
windbreak and sand fixation	$SR=SL_{q}-SL$	(4)	Where *SR* is the sand fixation rate, t/hm^2^; *SL_q_* is the potential soil wind erosion rate under bare soil conditions, t/hm^2^; *SL* is the actual soil wind erosion rate under Vegetation cover conditions, t/hm^2^; *Q_x_* is the sand flux at *x* (kg/m); *x* is the plot length, *Q_max_* is the maximum transfer amount, kg/m; *s* is the critical plot length (m); *WF* is the climate factor, which reflects the surface’s resistance to wind erosion; *EF* is the factor that represents the erodibility of the soil; *SCF* is the soil crust factor, *K’* is the surface roughness factor; and *COG* is the vegetation coverage factor.
	$Q_{x}=\frac{Q_{max}[1-e^{{\left(\frac{x}{s}\right)}^{2}}]}{x}$	(5)	
	$Q_{max}=109.8(WF\times EF\times SCF\times K^{\prime }\times COG)$	(6)	
	$WF=\frac{\sum_{i=1}^{N}WS_{2}{\left(WS_{2}-WS_{t}\right)}^{2}\times N_{d}\times \rho }{N\times g}\times SW\times SD$	(7)	Where *WS_2_* is the wind speed at 2 m, m/s, *WS_t_* is the critical wind speed at 2 m; *N* is the number of observations; *N_d_* is the number of test days; *ρ* is the air density, kg/m^3^, *g* is the acceleration due to gravity, m/s^3^; *SW* is the soil moisture factor; *SD* is the snow cover factor; *S_a_* is the sand content of the soil, *S_i_* is the silt content of the soil, *C_i_* is the clay content of the soil, *OM* is the organic matter content, and *CaCO_3_* is the calcium carbonate content.
	$EF=\frac{29.09+0.31S_{a}+0.17S_{i}}{100}+\frac{0.33\frac{S_{i}}{C_{i}}}{100}-\frac{2.95OM+0.95C_{a}CO_{3}}{100}$	(8)	
	$SCF=\frac{1}{1+0.0066{\left(C_{i}\right)}^{2}+0.021{\left(OM\right)}^{2}}$	(9)	
	$F_{c}=\frac{NDVI-NDVI_{soil}}{NDVI_{veg}-NDVI_{soil}}$	(10)	Fractional vegetation cover (FVC) data is calculated based on the theory of the cell binary model. In the formula, *F_c_* is the vegetation coverage; *NDVI_veg_* is the *NDVI* value at the 95% percentile; and *NDVI_soil_* is the *NDVI* value at the 5% percentile.
Water Yield (Zhang et al., 2022; Donohue et al., 2012)	$Y_{x,j}=(1-\frac{AET_{x,j}}{P_{x}})\cdot P_{x}$	(11)	Where *Y_x,j_* is the WY of grid *x* on land use type *j*, in mm; *AET_x,j_* is the actual evapotranspiration of grid *x* on land use type *j*, in mm; *P_x_* is the precipitation of grid *x*, in mm; *AWC_x_* is the available water content of vegetation; *Z* is the Zhang factor, which is an empirical parameter set to 3.6 in this study; *w_x_* is an empirical parameter; *MSD* is the maximum soil depth; *RD_x_* is the root depth; *PAWC* indicates the plant available water content; *SAN* is the sand content of the soil, *SIL* is the silt content of the soil, *CLA* is the clay content of the soil, and *OM* is the organic matter content of the soil.
	$\frac{AET_{xj}}{P_{x}}=\frac{1+w_{x}R_{xj}}{1+w_{x}R_{xj}+(\frac{1}{R_{xj}})}$	(12)	
	$AWC_{x}=MIN(MSD_{x},RD_{x})\times PAWC_{x}$	(13)	
	$R_{x}=\frac{\left(k_{x}\times ET_{0x}\right)}{P_{x}}$	(14)	
	$w_{x}=Z\frac{AWC_{x}}{P_{x}}$	(15)	
	$\begin{array}{c} PAWC=54.509-0.132\times SAN-0.03\\ \times {\left(SAN\right)}^{2}-0.55\times SIL-0.06\times {\left(SIL\right)}^{2}\\ -0.738\times CLA+0.007\times {\left(CLA\right)}^{2}-2.688\\ \times OM+0.501\times {\left(OM\right)}^{2} \end{array}$	(16)	

#### 2.3.3 Methodology for analyzing the synergistic relationship between trade-offs in ESs

##### 2.3.3.1 Partial correlation analysis

The initial step is to calculate the fundamental correlation coefficient (see “Equations 17–19”).


(17) $$r_{12(ij)}=\frac{\sum_{n=1}^{n}(ES1_{n(ij)}-\bar{ES1_{\left(ij\right)}})(ES2_{n(ij)}-\bar{ES2_{\left(ij\right)}})}{\sqrt{\sum_{n=1}^{n}{\left(ES1_{n(ij)}-\bar{ES1_{\left(ij\right)}}\right)}^{2}\sum_{n=1}^{n}{\left(ES2_{n(ij)}-\bar{ES2_{\left(ij\right)}}\right)}^{2}}}$$

Calculate the first-order partial correlation coefficient:


(18) $$r_{12\cdot 3(ij)}=\frac{r_{12(ij)}-r_{13(ij)}r_{23(ij)}}{\sqrt{\left(1-r_{13(ij)}^{2})(1-r_{23(ij)}^{2}\right)}}$$

Calculate the second-order partial correlation coefficient:


(19) $$r_{12\cdot 34(ij)}=\frac{r_{12\cdot 3(ij)}-r_{14\cdot 3(ij)}r_{24\cdot 3(ij)}}{\sqrt{\left(1-r_{14\cdot 3(ij)}^{2})(1-r_{24\cdot 3(ij)}^{2}\right)}}$$

Where *ES1* and *ES2* are two types of *ES*. *r* is the correlation coefficient between them. *i* and *j* represent the row number and column number in the raster data respectively. *n* is the number of years in the time series. *r_12(ij)_* is the simple correlation coefficient of *ES1* and *ES2* at pixel *ij*, where the two *ESs* are different. Similarly, *r_13(ij)_*, *r_23(ij)_*, *r_14(ij)_*, *r_24(ij)_* and *r_34(ij)_* can also be achieved. Under the condition of constant *ES1* or *ES2*, *r_12·3(ij)_* is the first-order partial correlation coefficient at pixel *ij*. Similarly, *r_14·3(ij)_*, *r_24·3(ij)_* and *r_12·34(ij)_* represents the second-order partial correlation coefficients at pixel *ij*, assuming that both *ES1* and *ES2* are constant. The significance of the relationship between ESs was evaluated using the t-test method.

##### 2.3.3.2 Root-mean-square deviation

Prior to calculating the RMSD, the data must be standardized in accordance with the following formula (see “Equations 20, 21”):


(20) $$ES_{std}=\frac{\left(ES_{i}-ES_{min}\right)}{\left(ES_{max}-ES_{min}\right)}$$

(21) $$\text{RMSD}=\sqrt{\frac{1}{\left(\text{n}-1\right)}\sum_{\text{i}=1}^{\text{n}}{\left({\text{ES}}_{\text{std}}-\bar{{\text{ES}}_{\text{std}}}\right)}^{2}}$$

Where, $ES_{std}$ represents the normalized ES value; $ES_{i}$ , $ES_{max}$ , $ES_{min}$ and $\bar{ES_{std}}$ are the ES value, maximum value, minimum value and average value, respectively; *n* represents the number of ESs.

#### 2.3.4 Driver analysis based on ML methods

##### 2.3.4.1 XGBoost model

XGBoost (eXtreme Gradient Boosting) is an ensemble learning algorithm based on gradient-boosted decision trees (Chen and Guestrin, 2016). Compared to the traditional gradient-boosted decision tree (GBDT) algorithm, XGBoost uses a second-order Taylor expansion of the loss function, improving the model’s ability to identify the optimal solution (Tian et al., 2024) (see “Equation 22”).


(22) $${\hat{y}}_{i}=\sum_{k=1}^{k}f_{k}(x_{i}),f_{k}\in F(i=1,2,\ldots ,n)$$

Where $F=\{f(x)=w_{q(x)}\}(q:R^{m}\to \{1,2,\ldots ,T\},w\in R^{T})$ is the classification and regression tree ensemble decision tree (CART), q is the sample mapped to the leaf node of the tree structure, *T* is the number of leaf nodes, and w is the fraction of leaf nodes.

The objective function of an XGBoost model includes an error function and a complexity function (see “Equation 23”).


(23) Obj=L+Ω

Where L is the error function and  Ω  is a complex function (see “Equations 24, 25”).


(24) $$L=\sum_{i=1}^{n}{\left(y_{i}-{\hat{y}}_{i}\right)}^{2}$$

(25) $$\Omega =\gamma T+\frac{1}{2}\gamma \sum_{j=1}^{T}w_{j}^{2}$$

Where  γT  is the regular term of *L1* and $\frac{1}{2}\gamma \sum_{j=1}^{T}w_{j}^{2}$ is the regular term of *L2*. On this basis, the model prediction value of order t is added, a new function of order t is added, and the $\;f_{t}(x_{i})\;$ approximate objective function is obtained by a second-order Taylor expansion (see “Equations 26, 27”):


(26) $$Obj^{t}\approx \sum_{i=1}^{n}[{\left(y_{i}-{\hat{y}}_{i}^{t-1}\right)}^{2}+2(y_{i}-{\hat{y}}_{i}^{t-1})f_{t}(x_{i})-h_{i}f_{t}^{2}(x_{i})]+\Omega$$

(27) $$\begin{array}{c} Obj^{\left(t\right)}\approx \sum_{i=1}^{n}[g_{i}w_{q(x_{i})}+\frac{1}{2}h_{i}w_{q(x_{i})}^{2}]+\gamma T+\frac{1}{2}\sum_{j=1}^{T}w_{j}^{2}\\ =\sum_{j=1}^{T}[(\sum_{i\in I_{j}}g_{i})w_{j}+\frac{1}{2}(\sum_{i\in I_{j}}h_{i}+\lambda )w_{j}^{2}]+\gamma T \end{array}$$

##### 2.3.4.2 Explanation of SHAP features

SHAP is an explainable ML method that interprets model predictions based on Shapley value theory, quantifying the contribution of each feature to the model output. A key advantage of this method is its ability to assess the impact of individual features on predictions for specific samples, distinguishing between positive and negative effects. The algorithmic process is described in the following section.

If the *i_th_* is a sample of *X_i_*, the first *j* is a sample of the *i_th_ X_ij_* feature, the model predicted value of the sample *y_i_*, and the *y_base_* baseline prediction of the entire model. The SHAP value is calculated using the following equation: (see “Equation 28”)


(28) $$y_{i}=y_{base}+f(X_{i_{1}})+f(X_{i_{2}})+\ldots +f(X_{i_{k}})$$

In this context, the term “*f(X_ij_)*” represents the SHAP value associated with feature “*Xij*.” To illustrate, *f(X_ij_)* signifies the contribution of feature 1 in sample *i* to the predicted value *y_i_*. Should the value of *f(X_j1_)* be positive, it can be concluded that the feature in question exerts a positive influence on the prediction. Should the value of *f(X_j1_)* be less than zero, it indicates that the feature has a negative impact on the prediction. The sensitivity of a feature to machine learning is gauged using the SHAP value, which quantifies the contribution of a feature indicator to a target variable after data normalization.

## 3 Results

### 3.1 Spatial and temporal changes in ESs

**Figure 3:**
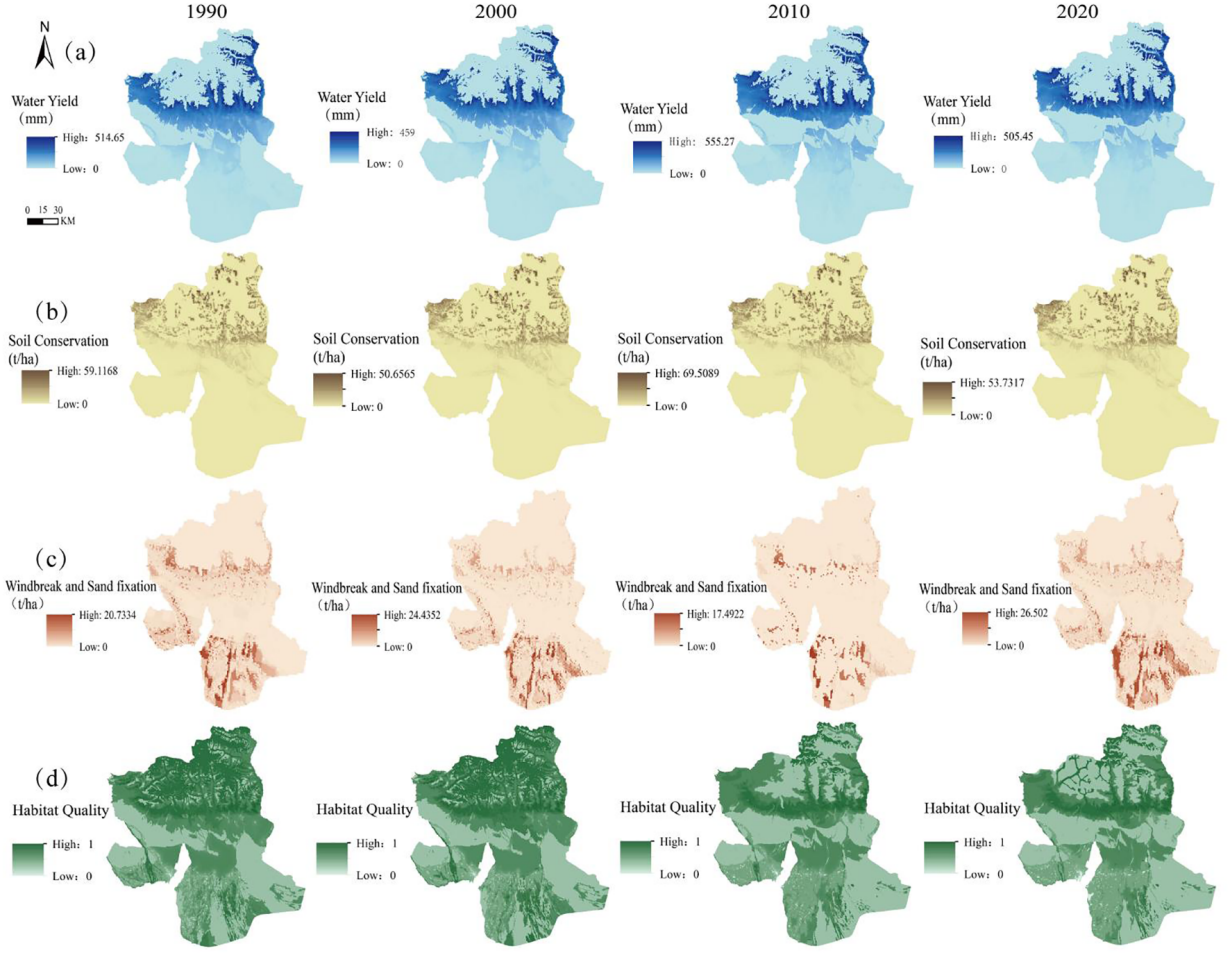
Spatial distribution of ESs. **(a)** Changes in the spatial distribution of WY from 1990 to 2020, **(b)** Changes in the spatial distribution of SC from 1990 to 2020, **(c)** Changes in the spatial distribution of WS from 1990 to 2020, **(d)** Changes in the spatial distribution of HQ from 1990 to 2020.

From 1990 to 2020, the total WY in Wensu County ranged from 812 million to 1.04 billion m³, with the average annual WY depth varying between 19.89 mm and 28.29 mm. Overall, it showed an inverted “N”-shaped trend: initially decreasing, then increasing, and finally decreasing again, with a slight overall decline. From 1990 to 2000, the mean annual WY decreased from 22.99 mm to 19.89 mm, then peaked at 28.29 mm in 2010, before declining again to 21.32 mm between 2010 and 2020 (**Table 3**). The spatial pattern of WY from 1990 to 2020 (**Figure 3a**) has undergone slight alterations, exhibiting a spatial distribution characterized by a decline from the center to the edges. Specifically, high-value areas are mainly located on the southern side of the northern mountains, while low-value areas are found in the central transitional zone and the southern plains.

**Table 3:** Temporal evolution of ESs.

Years	1990	2000	2010	2020
Average annual WY depth (mm)	22.99	19.89	28.29	21.32
SC per unit area (t/km^2^)	1440.28	1269.33	1718.51	1351.3
WS (t)	2.32×10^7^	2.4×10^7^	1.18×10^7^	3.11×10^7^
HQ	0.596	0.603	0.519	0.507

The SC per unit area in the study area for 1990, 2000, 2010, and 2020 was 1440.28 t/km², 1269.33 t/km², 1718.51 t/km², and 1351.3 t/km², respectively (**Table 3**). The change trend showed an inverted “N” shape, with a slight overall decrease, peaking in 2010. Regarding the spatial distribution (**Figure 3b**), the SC pattern showed a tendency toward stabilization from 1990 to 2020. The high-value areas are primarily located on the southern side of the northern mountainous area and extend to the east and west. They are distributed in a scattered manner, while the low-value areas are distributed in the southern plains and extend to the east and west.

The amount of WS in the research area in 1990, 2000, 2010 and 2020 was 2.32×10^7^t, 2.4×10^7^t, 1.18×10^7^t and 3.11×10^7^t, respectively (**Table 3**), showing an overall positive “N”-shaped increasing trend, with a significant rise from 2010 to 2020. In terms of spatial distribution (**Figure 3c**), the changes are more obvious. High-value areas are mainly located in the area extending westward from the southern plains and the intermediate transition zone, while the low-value areas are mainly located in the northern mountainous areas and the area extending eastward from the intermediate transition zone.

The average HQ index in the study area for 1990, 2000, 2010 and 2020 was 0.596, 0.603, 0.519 and 0.507, respectively (**Table 3**), showing an upward trend before declining, with the downward trend strengthening from 2010 to 2020. In terms of spatial distribution (**Figure 3d**), high-value areas are mainly distributed in the mountainous areas in the north and the plains in the south, while low-value areas are distributed in the areas extending to the east and west of the plains in the south. From 1990 to 2000, HQ remained generally stable. However, from 2000 to 2020, significant spatial distribution changes occurred, there was a significant decrease in HQ in the northern mountainous areas, and along with a gradual decline in HQ in the areas extending eastward from the southern plains.

### 3.2 Trade-offs and synergies of ESs

#### 3.2.1 Time scale trade-off synergy

**Figure 4:**
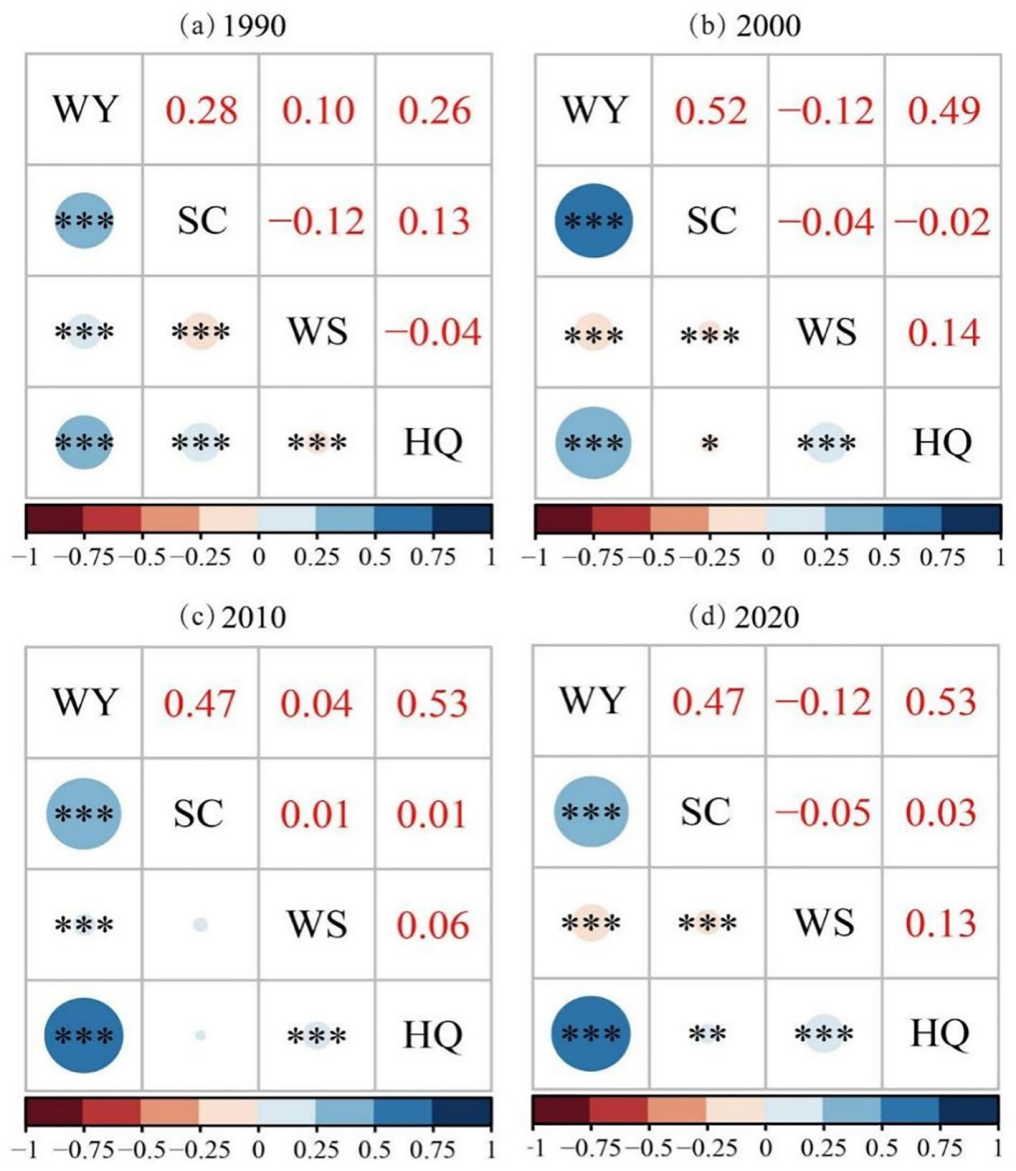
Partial correlation coefficient analysis. **(a)** Coefficient of bias analysis of ESs in 1990, **(b)** Coefficient of bias analysis of ESs in 2000, **(c)** Coefficient of bias analysis of ESs in 2010, **(d)** Coefficient of bias analysis of ESs in 2020. *p>0.1, **0.05<p<0.1, ***0.01<p<0.05.

The correlation coefficient map showing the relationship between ES (**Figure 4**) reveals that the trade-off and synergy dynamics among different ES groups remained relatively stable from 1990 to 2020. Specifically, the relationship between WY-SC demonstrated a clear synergistic pattern, with an initial increase, followed by a decline, and ultimately stabilizing over time. The strongest synergistic correlation occurred in 2000 (0.52), while the weakest was observed in 1990 (0.28). The relationship between WY-WS was synergistic in 1990 and 2010, although the synergy was weak. However, a trade-off relationship was showed in 2000 and 2020. On the other hand, the relationship between WY-HQ showed an increasing trend, followed by stabilization, with a significant synergistic correlation. The relationship between SC-WS exhibited a trade-off from 1990 to 2000, which gradually weakened, followed by a weak synergistic relationship in 2010, and a weak trade-off in 2020. The relationship between SC-HQ was synergistic in 1990, 2010, and 2020, but showed a weak trade-off in 2000. For WS-HQ, a weak trade-off was showed in 1990, while a synergistic relationship was seen from 2000 to 2020, with a trend of decreasing synergy followed by an increase.

#### 3.2.2 Spatial trade-off scale synergy

**Figure 5:**
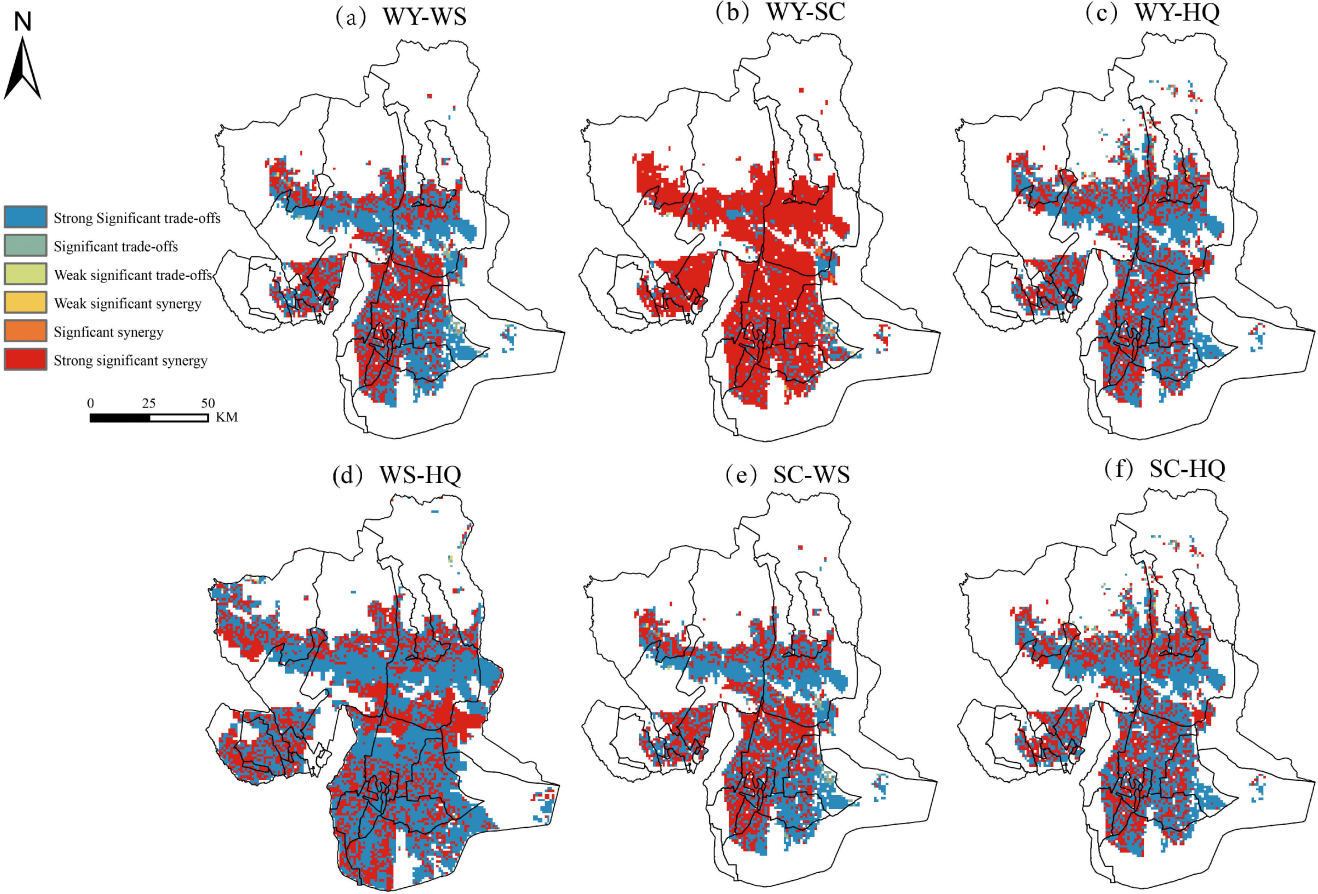
Spatial distribution of synergistic relationships between trade-offs in ESs. **(a)** Spatial distribution of trade-off synergies in WY-WS, **(b)** Spatial distribution of trade-off synergies in WY-SC, **(c)** Spatial distribution of trade-off synergies in WY-HQ, **(d)** Spatial distribution of trade-off synergies in WS-HQ, **(e)** Spatial distribution of trade-off synergies in SC-WS, **(f)** Spatial distribution of trade-off synergies in SC-HQ.

We analyzed the trade-off synergies between ESs in Wensu County from 1990 to 2020 pixel by pixel to compensate for the lack of intra-spatial differences in trade-off synergies. **Figure 5a** shows that the trade-off synergy relationship of WY-WS is relatively evenly distributed, with 55.33% of the pixels exhibiting a trade-off relationship, mainly concentrated in the southern part of the central transition zone and the area extending eastward from the southern plains. The pixel ratio of the collaborative relationship is 44.67%, primarily concentrated in the northern part of the central transition zone and the area extending westward from the southern plains. **Figure 5b** shows that WY-SC is predominantly manifested by synergy relationships, with 88.62% of the pixels exhibiting synergy, primarily concentrated in the central transition zone and the area extending westward from the southern plains. **Figure 5c** shows that WY-HQ is primarily manifested by trade-off relationships, with 56.85% of the pixels exhibiting trade-offs, primarily concentrated in the central transition zone and the area extending westward from the southern plains. **Figure 5d** shows that WS-HQ is primarily manifested by trade-off relationships, with 60.08% of the pixels exhibiting trade-offs, mainly concentrated in the central transition zone extending east and west, and the area extending westward from the southern plains. **Figure 5e** shows that the pixel ratio of the trade-off relationship for SC-WS is 54.33%, primarily concentrated in the southern part of the central transition zone and the eastern side of the southern plains. **Figure 5f** shows that SC-HQ is primarily manifested by the trade-off relationship, with 55.95% of the pixels exhibiting trade-offs, mainly concentrated in the southern part of the central transition zone and the area extending eastward from the southern plains.

#### 3.2.3 Intensity change of ESs trade-offs

**Figure 6:**
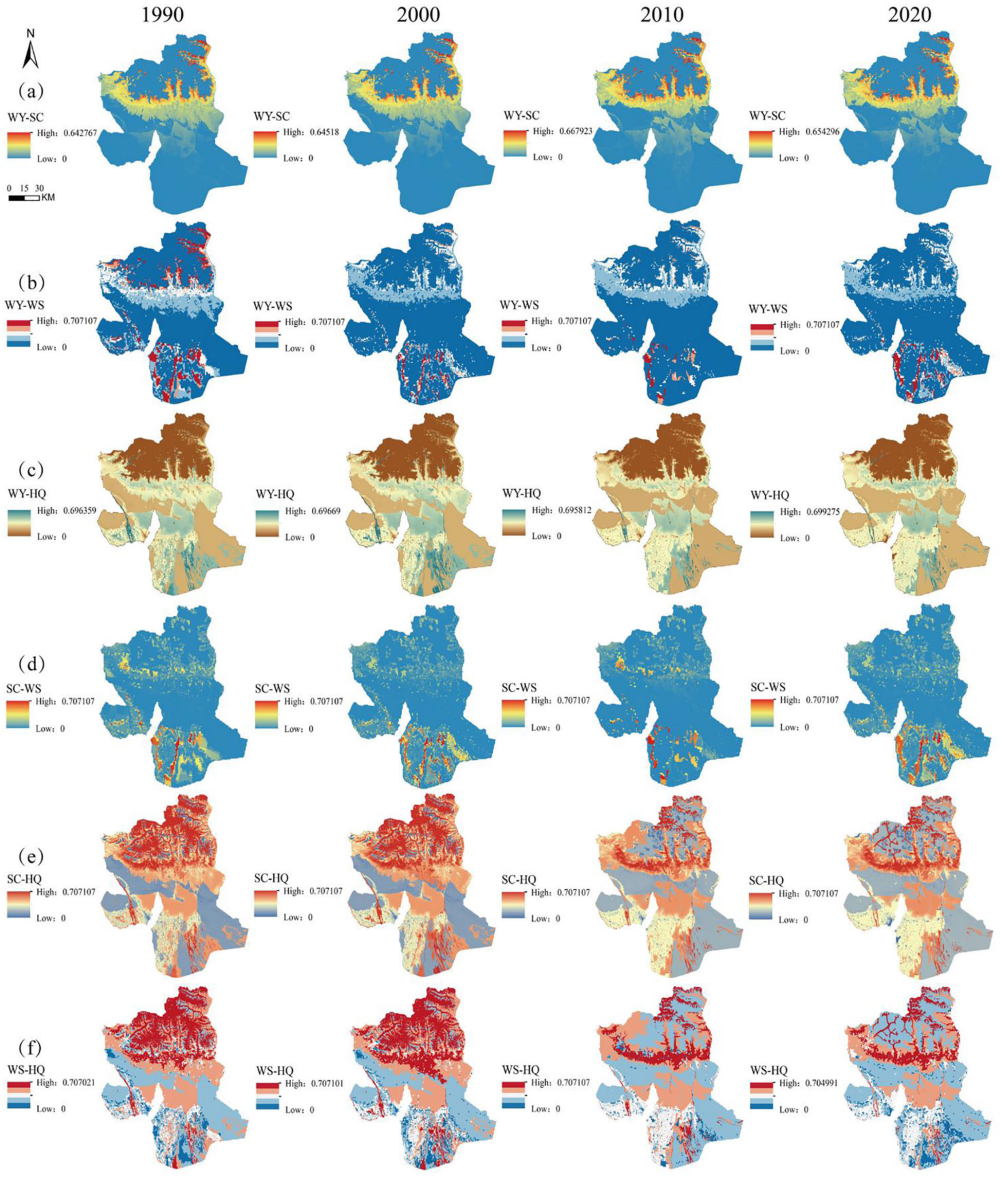
Spatial distribution of the intensity of trade-offs between ESs. **(a)** Spatial distribution of WY-SC trade-off intensities from 1990 to 2020, **(b)** Spatial distribution of WY-WS trade-off intensities from 1990 to 2020, **(c)** Spatial distribution of WY-HQ trade-off intensities from 1990 to 2020, **(d)** Spatial distribution of SC-WS trade-off intensities from 1990 to 2020, **(e)** Spatial distribution of SC-HQ trade-off intensities from 1990 to 2020, **(f)** Spatial distribution of WS-HQ trade-off intensities from 1990 to 2020.

**Figure 6** shows the spatial distribution of the trade-off between ESs in Wensu County. Compared to the other four trade-offs, the trade-off between SC-HQ and WS-HQ is notably stronger, while the trade-off between WY-SC is the weakest and is almost zero in the majority of areas, with the exception of the central transition zone, which indicates that WY-SC has a synergistic relationship in the majority of areas. From 1990 to 2020, the high-value area of WY-WS in the north gradually declined, while the high-value area in the southern plains showed a gradual increase, showing a striped distribution. The high-value areas of WY-HQ are scattered across the central transition zone and the southern side of the southern plains, while the low-value areas are concentrated in the northern mountainous regions. The high-value areas of SC-WS are mainly located on the southern side of the southern plains, forming a striped distribution pattern, and are distributed in dots in the northern mountainous areas. The spatial distribution of SC-HQ is similar to that of WS-HQ. From 1990 to 2000, the high-value area was concentrated in the northern mountainous region. However, from 2000 to 2020, there was a notable decrease in the trade-off intensity observed in this area. From 1990 to 2000, the low-value area was primarily situated on the eastern and western edges of the southern plain. From 2000 to 2020, in addition to the previously mentioned margins, a block-shaped low-value area emerged in the northern mountainous region.

### 3.3 Analysis of the drivers of the intensity of trade-offs in ESs

#### 3.3.1 Identification of key influencing factors

**Figure 7:**
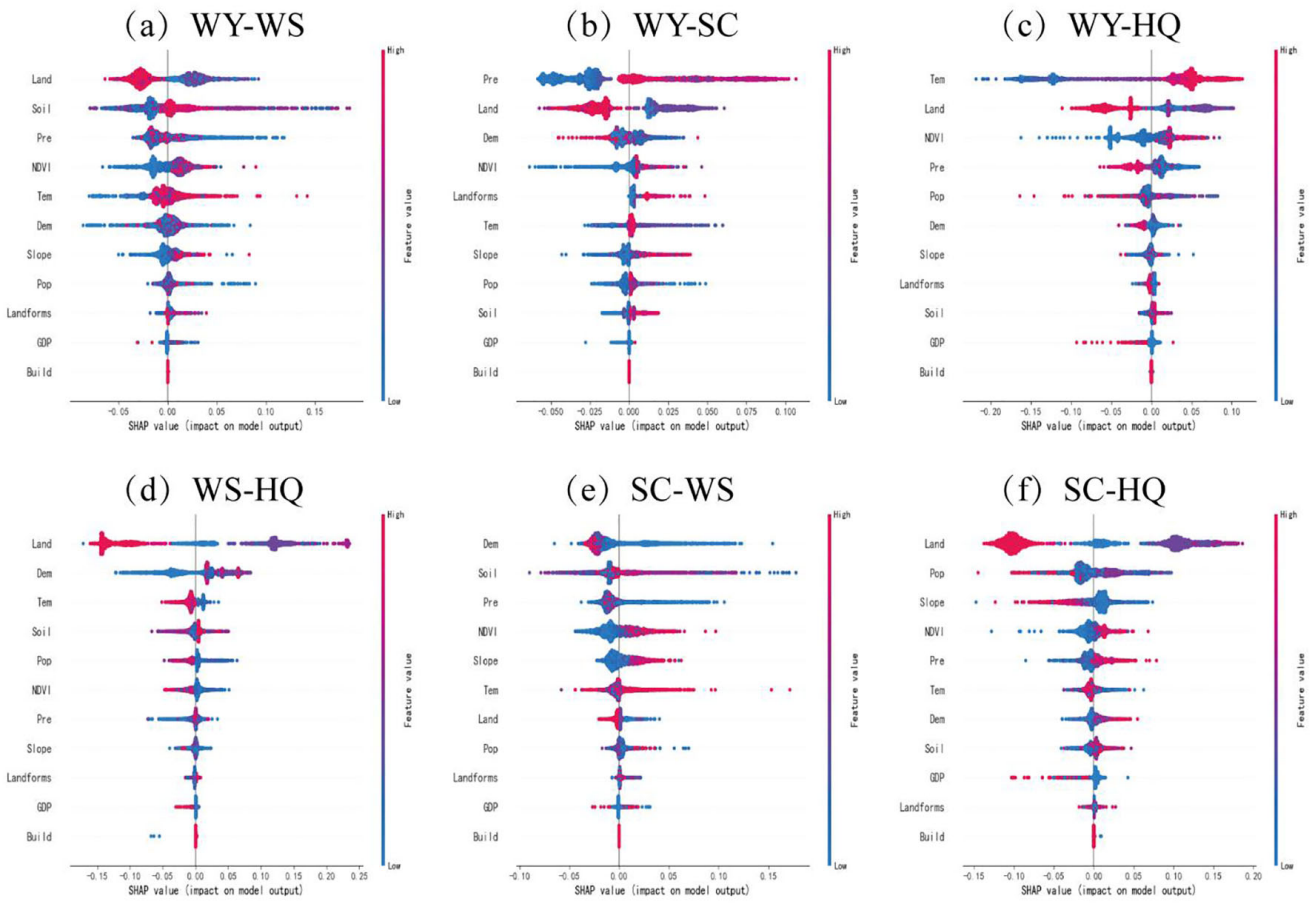
SHAP plot of drivers of trade-offs in ESs. **(a)** SHAP diagram of WY-WS drivers, **(b)** SHAP diagram of WY-SC drivers, **(c)** SHAP diagram of WY-HQ drivers, **(d)** SHAP diagram of WS-HQ drivers, **(e)** SHAP diagram of SC-WS drivers, **(f)** SHAP diagram of SC-HQ drivers.

The study used a combination of XGBoost and SHAP methods to analyze the relative importance of each driver in the ESs trade-off relationship in Wensu County and the direction of its movement (**Figure 7**). The results show that in WY-WS, Land is the most important driving factor, followed by Soil. In terms of the direction of movement, Soil, NDVI, Tem, Slope, and Landforms promote the ESs trade-off, while Land and GDP inhibit the ESs trade-off. In WY-SC, Pre is the most important driving factor, followed by Land. Among these, Pre, NDVI, Landforms, Slope, Soil, and GDP promote the ESs trade-off, while Land and Dem inhibit the ESs trade-off. In WY-HQ, Tem is the most important driving factor, followed by Land. Among these, Tem, NDVI, and Soil promote the ESs trade-off, while Land, Pre, Pop, Dem, Slope, and GDP have a suppressive effect on the ESs trade-off. In WS-HQ, Land is the most important driving factor, followed by Dem. Among these, Dem, Soil, and Landforms promote the ESs trade-off, while Land, Tem, Pop, NDVI, and GDP have a suppressive effect on the ESs trade-off. In SC-WS, Dem is the most important driving factor, NDVI and Slope promote the trade-off of ESs, while Dem, Land and GDP inhibit the trade-off of ESs. In SC-HQ, Land is the most important driving factor, NDVI, Pre, Dem and Soil promote the trade-off of ESs, while Land, Pop, Slope and GDP inhibit the trade-off of ESs.

#### 3.3.2 Nonlinear relationship between key influencing factors and effectiveness threshold

**Figure 8:**
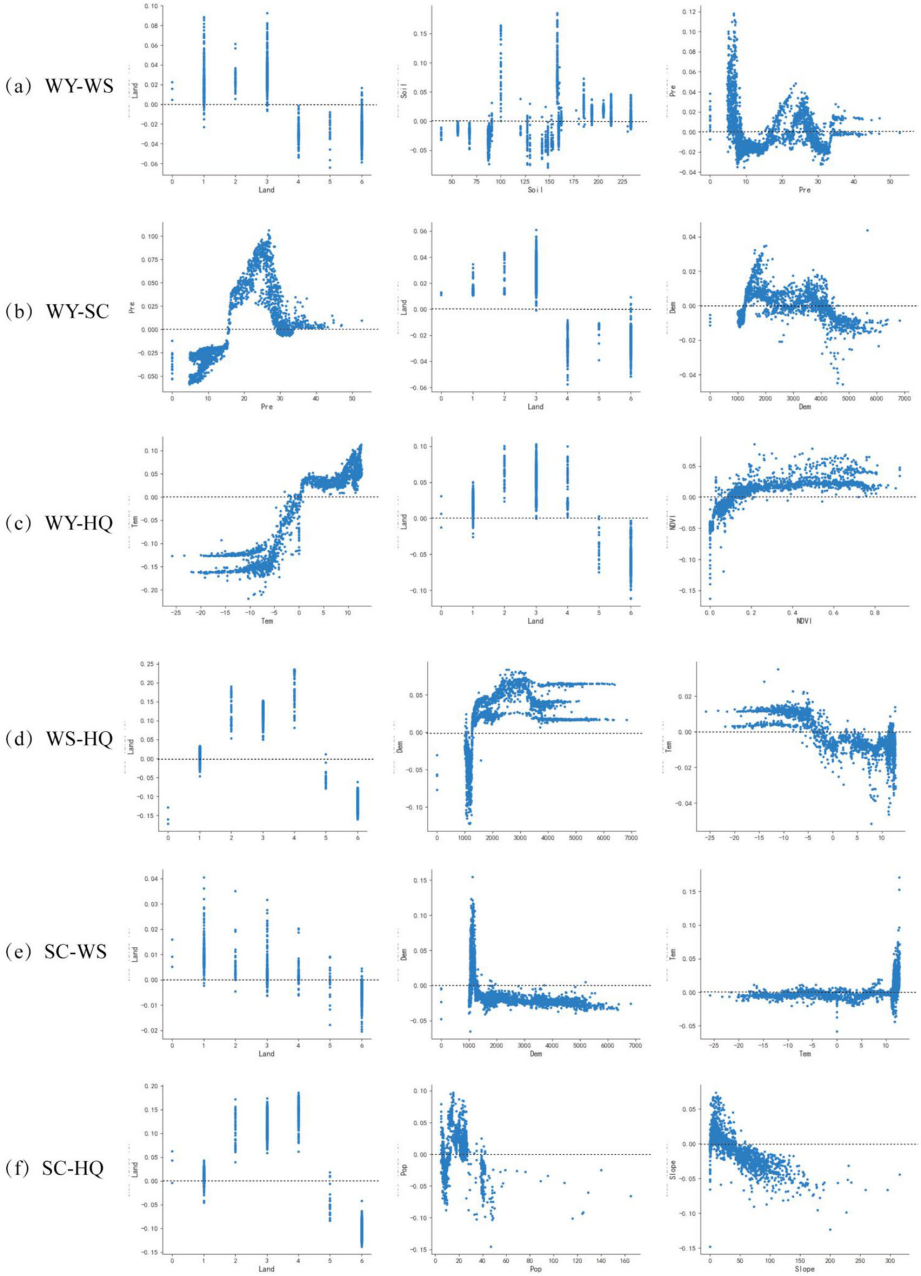
Nonlinear characteristics of ES trade-off drivers. **(a)** Nonlinear characteristics of WY-WS trade-off drivers, **(b)** Nonlinear characteristics of WY-SC trade-off drivers, **(c)** Nonlinear characteristics of WY-HQ trade-off drivers, (d) Nonlinear characteristics of WS-HQ trade-off drivers, **(e)** Nonlinear characteristics of SC-WS trade-off drivers, **(f)** Nonlinear characteristics of SC-HQ trade-off drivers.

In general, varying degrees of autocorrelation exist between the drivers of ES trade-offs, complicating the analysis of these factors individually. The SHAP plot method provides a more effective way to isolate the impact of specific drivers and to identify trends in ES trade-offs driven by these factors (**Figure 8**). This study focuses on the top three key drivers that significantly impact on ES trade-offs and provides a detailed analysis. In WY-WS, the trade-off is promoted when Land is cropland, forest land, or grassland, and suppressed when Land is water, construction land, or unused land. When Soil is swamp soil, salty soil, irrigated soil, frozen soil, or cold calcareous soil, it promotes trade-offs, while gray-brown soil, brown soil, brown desert soil, stony soil, meadow soil, and flooded soil inhibit trade-off. Pre exhibits a clear threshold effect on the ESs trade-off. When Pre is less than 7 mm or between 16 mm and 26 mm, it positively contributes to the trade-off, promoting it. Conversely, when Pre is between 7 mm and 16 mm or between 26 mm and 34 mm, the effect of Pre on trade-offs is suppressed. In WY-SC, Pre promotes trade-offs when it is between 17 mm and 29 mm. Similarly, a specific altitude range has a significant impact on the ESs trade-off. Altitudes between 1,200 m and 3,600 m promote the trade-off. The trade-off is promoted when Land is cropland, forest land, or grassland, and suppressed when Land is water, construction land, or unused land. The effect of Dem on the trade-off is suppressed when Dem is below 1,200 m or above 3,600 m. In WY-HQ, when Tem is above -2°C, the effect on the trade-off is facilitated. When Tem is below -2°C, it inhibits the trade-off. When Land is cropland, forest land, grassland, or water area, which facilitates trade-off, while land is shown as construction land or unused land, which inhibits trade-off. NDVI also shows a significant threshold effect on the ES trade-off, with a threshold value of 0.08. When NDVI exceeds 0.08, it favors the trade-off, while values below 0.08 indicate that the trade-off is suppressed. In WS-HQ, Land classified as cropland, forest land, grassland, or water area promotes the trade-off, while construction land or unused land suppresses it. It promotes trade-offs when Dem is greater than 1200m. It promotes trade-offs when the temperature is below -5°C and inhibits trade-offs when it is above -5°C. In SC-WS, when Land is cropland, forest land, grassland, or water area, the trade-off is promoted, while construction land or unused land suppresses it. When Dem is between 1000m and 1300m, the trade-off is promoted, and when Dem below 1000m above 1300m, the trade-off is suppressed. Tem between -12°C and -1°C or above 5.5°C promotes trade-off, while Tem below -12°C and between -1°C and 5.5°C suppresses trade-off. In SC-HQ, when land is cropland, forest land, grassland, or water, trade-offs are facilitated, while construction land or unused land suppresses them. Pop between 18.5 people/km^2^ and 20 people/km^2^ promote the trade-off, while Pop below 18.5 people/km^2^ or above 20 people/km^2^ suppress it. A slope of less than 50°promotes the trade-off, while a slope greater than 50°suppresses it.

## 4 Discussion

### 4.1 Analysis of ESs assessment

Studies have shown that the high-value areas of WY, WS, SC and HQ in Wensu County are primarily located in the large forest and grassland area of the northern and central regions, which is consistent with the findings of Wang et al. (2020) and Zhang et al. (2023). This phenomenon is primarily attributed to the relatively high precipitation, which creates an ideal habitat for species and enhances SC and WS ability (Li S, et al., 2021). Therefore, conserving mountain vegetation and water resources is crucial for the sustainable development of Wensu County, and land use changes directly influence the temporal and spatial distribution patterns and evolution of ES functions. ESs vary by region. WY is assessed based on data such as rainfall and evapotranspiration, but it does not account for meltwater from glaciers and snow, potentially leading to an underestimation of WY. Precipitation is the primary factor influencing WY, significantly affecting the quantity of water resources and terrestrial hydrological processes (Ziadat and Taimeh, 2013). Additionally, SC is closely linked to land use type and climate. Amid global warming (Chen et al., 2020), the water cycle in the study area has intensified, leading to increased precipitation and snowmelt in the mountains. 2010 was a year of abundant rainfall in Wensu County (Sannigrahi et al., 2019), and the increase in vegetation cover enhanced SC capacity, with WY and SC reaching their peak values during the study period. After 2010, WY and SC declined due to the effects of climate change and changes in land use, with the overall trend slightly weakening.

WS is an extremely important ES in Wensu County, with changes in vegetation and climatic factors being the primary drivers of its variation. Forests have a greater ability to reduce wind speed and regulate climate than grasslands and farmlands. However, the study area has a relatively small forest area and a larger grassland area. In 2010, the amount of sand fixation reached a minimum. Wensu County is located in an arid to semi-arid region, with precipitation concentrated in the summer and windy days in the spring. The effect of summer precipitation on soil and vegetation is not enough to control soil erosion, which mainly occurs in spring, Additionally, 2010 saw abundant rainfall, and the heavy precipitation may have washed away topsoil, particularly on sloping land or areas with exposed soil. The impact of precipitation was strong and could cause soil particles to flow with the water, resulting in loss of sand. However, in the past two decades, global warming has led to a decrease in wind speed in the study area, resulting in an overall increase in WS.

The overall HQ is on a downward trend, which is closely related to the development status of the research area. Currently, the development of Wensu County is characterized by low-quality growth, evidenced by the rapid expansion of construction land, which has encroached on large areas of cultivated land. This, in turn, has increased pressure on ecological resources, leading to a significant reduction in both forest and unused land. The intensification of human activities has driven rapid urbanization and agricultural expansion, is a key factor in the decline of regional HQ.

### 4.2 Changes in ESs trade-offs and synergies

The study results show that WY-SC and WY-HQ have remained in synergy from 1990 to 2020, as they are closely linked to vegetation cover and precipitation. Precipitation promotes vegetation growth and improves HQ and SC capacity, resulting in WY-SC and WY-HQ showing the same or lower values, and therefore a strong synergistic correlation. WS is influenced by natural factors such as soil and terrain, as well as climatic factors like temperature and wind speed, leading to a large difference in its relationship with the other three services.

The results of changes in the region show that, except for the synergy maintained by WY-SC, the other ES combinations mainly show spatial trade-offs. Due to the multiple influences of natural and social factors, the trade-off synergy of ESs shows obvious spatial heterogeneity, which reflects the comprehensiveness and complexity of ecological processes (Niu et al., 2022; Cui et al., 2023). Quantifying the spatial pattern of ESs trade-offs in Wensu County over the past two decades using RMSD reveals that the intensity of trade-offs WY-WS and SC-WS has generally increased. This is attributed to the increase in precipitation, which enhances vegetation cover and, in turn, increases WY and SC. However, the intense impact of heavy precipitation can erode the soil’s surface layer, causing soil particles to be carried away with the water, which leads to soil erosion and reduces the ability to prevent sandstorms. The overall decrease in the strength of trade-offs among other ESs suggests that conflicts between different services have diminished, indicating that the ecological compensation mechanism in the area has had some success. The value of the trade-off between WY-SC is minimal, and except for areas with high vegetation cover in the central transition zone, the trade-off strength in other areas is almost zero, which also indicates that there is a synergistic relationship between WY-SC in most areas.

### 4.3 Drivers of ESs

Understanding the driving mechanisms behind changes in ES trade-offs and identifying key influencing factors is critical for the protection of vulnerable ecosystems. This study marks the first application of XGBoost-SHAP to ecologically fragile areas, showcasing its potential to reveal hidden patterns and relationships that might otherwise remain undetected. The results provide novel and actionable insights for ecosystem managers and policymakers, facilitating the development of more precise, region-specific management strategies.

The complex relationships among ESs trade-offs are influenced by both natural and human factors (Fang et al., 2024). The results of the XGBoost-SHAP model in this study show that the importance of influencing factors varies among different combinations of ESs, and exhibits a significant threshold effect, which is consistent with the findings of Wang et al. (2024) and Tian et al. (2024). In WY-WS, WS-HQ, and SC-HQ, land is the most important factor influencing the strength of trade-offs. Land can increase the trade-off between water and soil services when precipitation is below the critical value by changing land surface roughness and water infiltration capacity, while alleviating HQ conflicts by enhancing soil carbon sequestration capacity in areas with high vegetation coverage, especially in the ecologically fragile zone of arid areas. Therefore, an important breakthrough to improve the strength of trade-offs is to strategically determine the scope of construction land expansion from the perspective of land use, reasonably develop unused land, and reduce the destruction of forest land, grassland, and other natural landscapes. In WY-SC, Pre is the factor with the largest contribution rate and shows a significant non-linear change, with trade-offs being promoted when Pre is between 17 mm and 29 mm. Meanwhile, in WY-HQ, Tem is the factor with the largest contribution rate, and when Tem is greater than -2°C, it has a positive effect on trade-off. When Tem is less than -2°C, it suppresses the trade-off. The synergistic relationship between temperature on WY and HQ showed a significant threshold effect, and the opposition between soil moisture conservation mechanism and evapotranspiration water consumption during the freezing period explained why the WY-HQ synergy could be maintained in the cold and arid mountainous areas in the north, while the conflict in the southern plains intensified under the background of warming. Therefore, climate change may affect the strength of the trade-off between WY-HQ and WY-SC by changing Pre and Tem. These nonlinear mechanisms suggest that the service trade-off management in ecologically fragile areas needs to break through the traditional linear programming thinking and focus on the threshold response and interaction paths of key drivers. For example, priority should be given to protecting the high vegetation cover zone in areas with severe precipitation fluctuations, implementing habitat corridor projects in the thermal transition zone, and breaking the vicious circle of “population-arable land-ecology” through land-use zoning. In conclusion, climate change must be closely monitored and responsive measures must be taken to improve the efficiency of resource use, especially ecological efficiency, in the management of ES in Wensu County. Changing the strength and direction of driving factors can significantly improve the targeted ESs and pay more attention to the implementation of ecosystem restoration policies.

The application of XGBoost-SHAP in this study also sets a strong precedent for its use in future research, offering a powerful tool for understanding and managing ES trade-offs in vulnerable ecosystems globally. The model’s nonlinear predictive capabilities, combined with its interpretability, make it a robust framework for exploring ESs interactions and guiding effective, sustainable management practices. Through this approach, the model facilitates the design of tailored, targeted policies that respond to the specific ecological needs of regions, paving the way for more effective ecosystem restoration and conservation strategies.

### 4.4 Related policies and recommendations

The ecosystem of Wensu County is characterized by diverse features, including significant altitude variations, an uneven distribution of land resources, and distinct regional differences in the human-environment relationship. These factors contribute to the uneven distribution of ESs to a certain extent. The study results indicate significant temporal and spatial variations in ESs across Wensu County, with Land, Pre, and Tem being the primary factors influencing the strength of trade-offs in these services. Therefore, it is crucial for the government to fully consider the unique characteristics of the terrain and landforms in both northern and southern regions, as well as the threshold and non-linear features of the influencing factors when formulating policies, which will help formulate targeted ecosystem management policies (Wang and Xu, 2023). Based on the characteristics of ES trade-offs in different regions, this study provides policy recommendations for Wensu County.

1. The northern mountainous region is characterized by a complex topography, steep slopes and poor site conditions, which have resulted in the significant prevalence of soil erosion and ecological degradation. Moreover, the region experiences notable temporal and spatial variations in HQ. Beyond topographic influences, climatic factors—such as seasonal fluctuations in precipitation—and soil attributes, including water retention capacity and fertility, play critical roles in determining the ecosystem’s stability and functionality. Consequently, the primary goal of ecological management should focus on soil and water conservation through a comprehensive approach. This includes restoring closed forest land and grassland, cultivating multi-layered and diverse vegetation communities, revitalizing regional biodiversity, and enhancing the area’s soil and water conservation capacity, thereby mitigating land degradation and supporting the overall recovery of the ecological environment.
2. The southern plains region is characterized by relatively flat terrain. However, the region is confronted with two significant challenges: a high population density and limited land resources. These issues have led to low land productivity and restricted growth in grain yields from cropland. This has further exacerbated food security concerns and placed considerable pressure on the supply of ES functions. It is therefore recommended that ES supply be optimized through the protection, management, restoration, and conservation of natural vegetation, alongside the development of characteristic plants to improve the cultivation structure, particularly in areas with high soil fertility and favorable water and temperature conditions. In such areas, priority should be given to the cultivation of cash crops. This will not only enhance the efficiency of agricultural production, but also alleviate the pressure on cropland resources and provide local farmers with a more lucrative source of income. In conclusion, In the process of land use, it is essential to consider the implementation of a diversified agricultural production system. This system should combine sustainable agricultural techniques, such as crop rotation and fallow farming, with the objective of achieving a balance between agricultural output and ecological conservation.

### 4.5 Limitations of the study and future research directions

This study has certain limitations due to differences in data sources and model parameter settings. In addition, when different models are used for evaluation in this study, the accuracy of the results may also be affected due to the different accuracy of some data. Therefore, Future research on trade-offs and synergies should focus on the comprehensive assessment and parameter setting of ESs. Additionally, it is essential to investigate the complex mechanisms through which ecological and social factors influence ES trade-offs, ensuring that regional differences and the contributions of driving factors are fully incorporated into policy development.

## 5 Conclusion

This study takes Wensu County as the research area. According to the local ecological vulnerability characteristics, four ESs, WY, SC, WS and HQ, from 1990 to 2020 are selected for quantification. The relationships between ESs are assessed using partial correlation analysis and the pixel-by-pixel method. The strength of ES trade-offs is analyzed using RMSD, while explainable ML algorithms are employed to identify the key drivers influencing these trade-offs. The results show that:

1. From 1990 to 2020, Wensu County WY and SC showed an inverted “N”-shaped downward trend. The average annual WY decreased from 22.99 mm to 21.32 mm, and the SC per unit area decreased from 1440.28 t/km^2^ to 1351.3 t/km^2^. In contrast, the total amount of WS showed a positive “N”-shaped increasing trend, from 2.32×10^7^t to 3.11×10^7^t. Meanwhile the average HQ index showed an increasing trend before decreasing, with values of 0.596, 0.603, 0.519, and 0.507, respectively.
2. The relationships between WY-WS, WY-HQ, WS-HQ, SC-WS, and SC-HQ are primarily characterized by trade-offs, with trade-off pixel ratios of 55.33%, 56.85%, 60.08%, 54.33%, and 55.95%, respectively. However, the WY-SC relationship is primarily characterized by synergistic interactions, with a pixel ratio of 88.62%. Moreover, the trade-off strength between SC-HQ, WY-HQ, and WS-HQ is significantly higher, while the trade-off strength between WY-SC is the lowest, indicating that WY-SC has synergistic relationships in most regions.
3. The importance of the drivers of each group of ES trade-off effects is ranked differently, and the contribution of the same driver in different ES trade-off strength relationships is also different. Additionally, the direction of movement of different drivers in different ES trade-off strength relationships is also different. Each group of ES trade-off effects shows a nonlinear change in response to the driver, and the threshold of the same driver in different trade-off strengths is also different.

## Data Availability

The datasets presented in this study can be found in online repositories. The names of the repository/repositories and accession number(s) can be found in the article/supplementary material.
